# Wogonin inhibits in vitro herpes simplex virus type 1 and 2 infection by modulating cellular NF-κB and MAPK pathways

**DOI:** 10.1186/s12866-020-01916-2

**Published:** 2020-07-28

**Authors:** Ying Chu, Xiaowen Lv, Longfeng Zhang, Xingli Fu, Siwei Song, Airong Su, Deyan Chen, Lianhong Xu, Yongfang Wang, Zhiwei Wu, Zhihua Yun

**Affiliations:** 1 Clinical Laboratory, Wujin Hospital Affiliated with Jiangsu University, Wujin Clinical College of Xuzhou Medical University, Changzhou, 213017 China; 2 Department of Pediatrics, Affiliated Hangzhou First People’s Hospital, Zhejiang University School of Medicine, Hangzhou, 310006 China; 3 Clinical Laboratory, Affiliated Hospital of Jiangsu University, Zhenjiang, 212013 China; 4 Health Science Center, Jiangsu University, Zhenjiang, 212001 China; 5 Department of Cardiology, Renmin Hospital of Wuhan University, Wuhan, 430060 China; 6 Central Laboratory, The Second Affiliated Hospital of Nanjing Medical University, Nanjing, 210003 China; 7 Center for Public Health Research, Medical School, Nanjing University, Nanjing, 210093 China

**Keywords:** Wogonin, Herpes simplex virus (HSV), Antiviral activity, Nuclear factor κB (NF-κB), Mitogen-activated protein kinase (MAPK)

## Abstract

**Background:**

Wogonin, a natural flavonoid-like chemical compound, exhibits anti-inflammatory, antitumor, antiviral, neuroprotective, and anxiolytic effects by modulating a variety of cellular signaling pathways including PI3K-Akt, p53, nuclear factor κB (NF-κB), mitogen-activated protein kinase (MAPK) pathways. In this study, its antiviral effect against herpes simplex virus (HSV) type 1 and 2 (HSV-1 and HSV-2) replication was investigated.

**Results:**

Wogonin suppressed HSV-2-induced cytopathic effect (CPE) and reduced viral mRNA transcription, viral protein synthesis, and infectious virion particle titers in a dose-dependent manner. A time-of-drug-addition assay demonstrated that wogonin acted as a postentry viral inhibitor. Wogonin also significantly reduced HSV-induced NF-κB and MAPK pathway activation, which has previously been demonstrated to be important for viral replication.

**Conclusions:**

Our results suggest that the anti-herpes effect of wogonin may be mediated by modulation of cellular NF-κB and JNK/p38 MAPK pathways and imply that wogonin may be useful as an anti-HSV agent.

## Background

Herpes simplex virus (HSV) types 1 and 2 (HSV-1 and HSV-2, respectively), the two serotypes of the *Herpesviridae* family [1], are the most prevalent human pathogens that cause watery blisters on the skin or mucosae. HSV-1 infects mainly oral epithelial tissues and can cause herpes labialis and devastating encephalitis [2], In contrast, HSV-2 infects mainly the genital mucosa, and HSV-2 infection has been shown to be a risk factor for certain sexually transmitted diseases, such as Acquired Immune Deficiency Syndrome (AIDS) [3–5]. Due to the unavailability of cures for or vaccines against HSV infection, antiviral treatment is the only way to suppress primary and recurrent infection in clinical. Approved anti-HSV medications such as acyclovir, penciclovir and valacyclovir are specific inhibitors of herpesvirus DNA polymerase, but clinical evidence has shown that their use gives rise to the emergence of drug resistant mutants [6]. Therefore, identification of novel agents or compounds with different anti-HSV mechanisms is urgently needed. Among these, certain natural products from traditional Chinese herbs are alternative sources of such compounds.

Wogonin (5, 7-dihydroxy-8-methoxyflavone, MW. 284.27), an O-methylated flavonoid compound, is originally derived from the root of the traditional Chinese medical herb Huang-Qin (*Scutellaria baicalensis* Georgi). *S. baicalensis* has been widely used for the clinical treatment of various conditions, such as hepatitis, hypertension, diarrhea, the common cold, and inflammation [7]. Wogonin, one of the bioactive constituents in *Scutellaria* radix extract, has been found to exert anti-inflammatory, anti-tumor, anti-viral, neuroprotective, and anxiolytic effects in recent relevant studies [7]. Previous studies on its antiviral activity focused mainly on its inhibitory effects on respiratory syncytial virus (RSV) [8], hepatitis B virus (HBV) [9, 10], and varicella-zoster virus (VZV) [11] replication. Wogonin exerts anti-HBV activity by inhibiting secretion of HBV antigen HBsAg and reducing the levels of HBV DNA in vitro, as confirmed in vivo in animal models (duck HBV [DHBV]-positive ducks and HBV-transgenic mice) [9, 10]. Although the antiviral activity of wogonin has been confirmed, its precise inhibitory mechanism has not been fully elucidated.

In our previous work, we screened 600+ natural compounds obtained from Chinese National Compound Library for discovering the novel anti-HSV agents and found that wogonin inhibited HSV-induced cytopathic effect (CPE) under light inverted microscope. In this study, we would investigate the possible mechanism of action of wogonin. Our evidences would shed light on anti-HSV activity of wogonin and its possibility as a potential candidate agent in clinical application.

## Results

### Wogonin inhibits HSV-1/2 replication in vitro

**Fig. 1:**
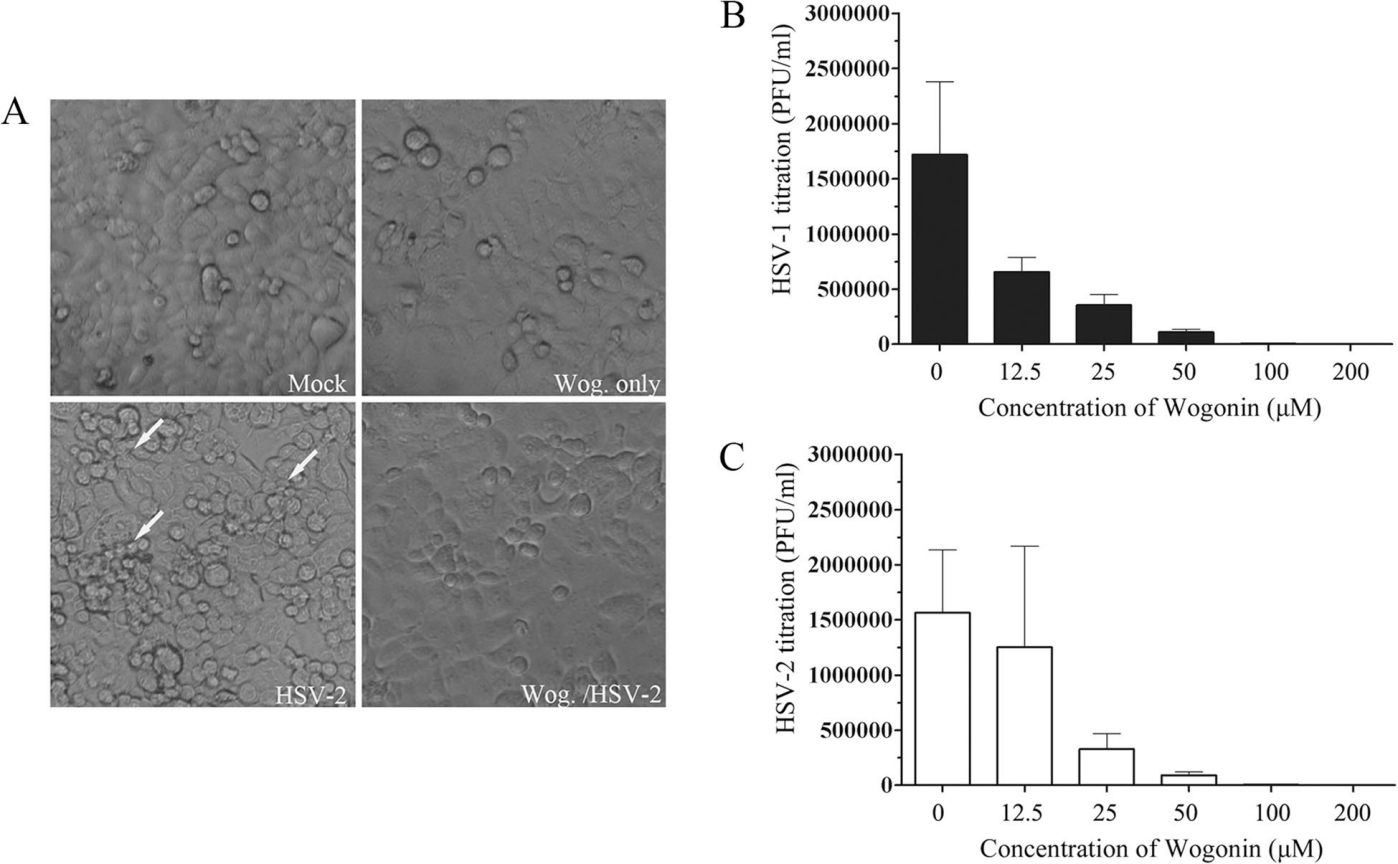
Wogonin inhibited HSV-induced CPEs and viral infectious particle formation in vitro. **a** Wogonin inhibited HSV-2-induced CPE development. HEC-1-A cells were mock-infected or infected with HSV-2 (G) (MOI = 1) in the presence or absence of wogonin (100 μM). Images were captured under an optical inverted microscope 24 h p.i. **b** and **c** Wogonin inhibited the formation of intracellular HSV-1/2 infectious viral particles. HEC-1-A cells were pretreated with serial concentrations of wogonin prior to infection with HSV- 1 (HF) or HSV-2 (G) (MOI = 1) for 24 h. The infectious viral particles were released by freeze and thaw cycles, and viral infectivity was titrated via measurement of the PFUs as described. The titers of HSV-1/2 infectious virions are shown as the means ± the SDs from three separate experiments

Under light microscope observation, we found that wogonin inhibited significant visible HSV-2-induced CPE in human endometrial HEC-1-A monolayers. HEC-1-A cells were preincubated with 100 μM wogonin for 30 min, and then infected with HSV-2 (G) (multiplicity of infection [MOI] = 1) for 24 h. It was shown that wogonin might prevent HSV-2 infection (Fig. 1a). To better understand the suppressive effect on infectious viral particle formation, HEC-1-A cells were infected with HSV-1 (HF) and HSV-2 (G) (MOI = 1) and treated with serial concentrations of wogonin. At 24 h post infection (p.i.), intracellular infectious viral particles were released via freeze and thaw cycles, and the virion yields were determined by titration of the plaque forming units (PFUs) in Vero cells. The results illustrated that wogonin could suppress production of both HSV-1 (Fig. 1b) and HSV-2 (Fig. 1c) virions in a dose-dependent manner, which in turn could block viral life cycle.

**Fig. 2:**
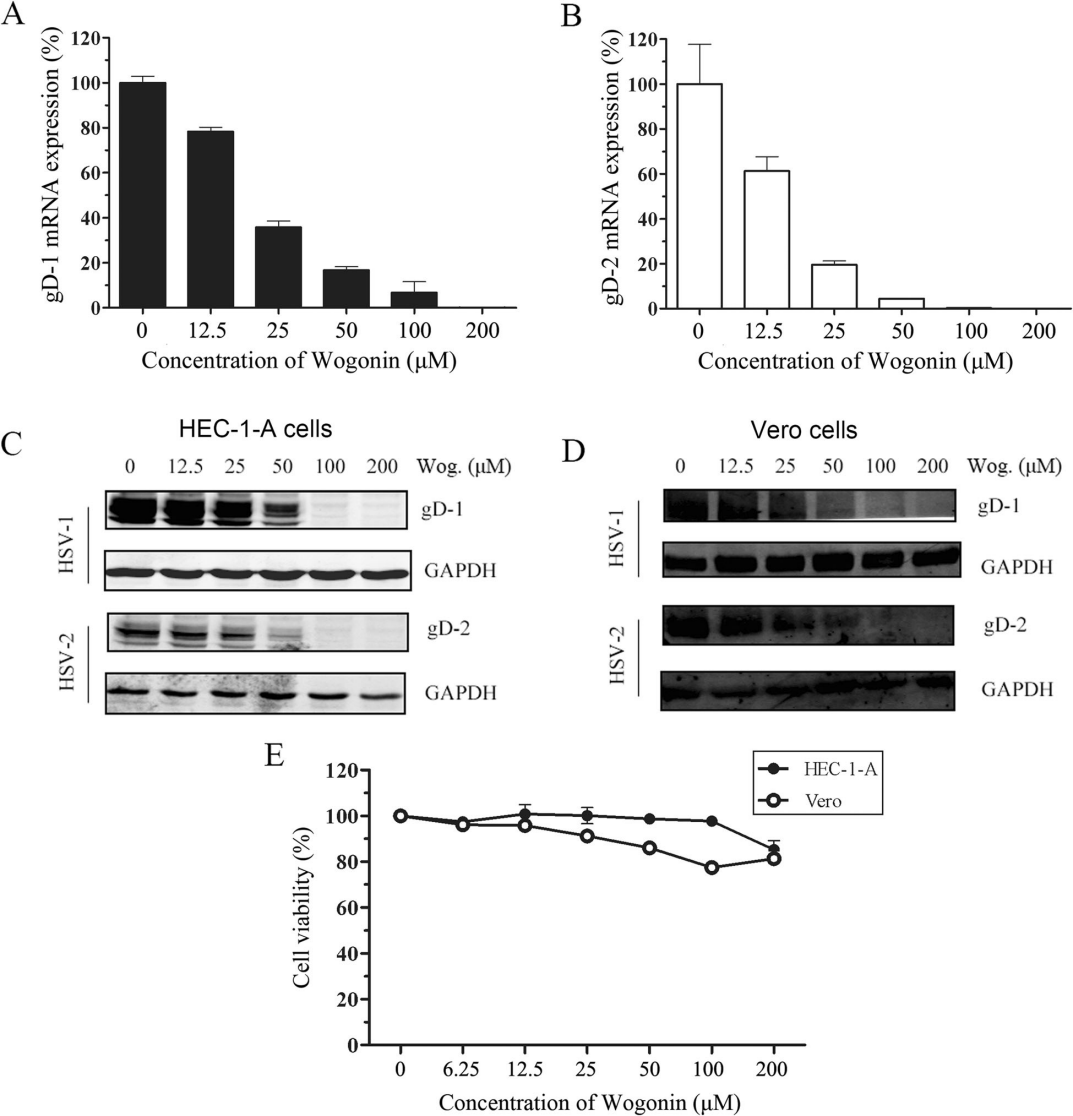
Wogonin inhibited HSV L gene expression. **a** and **b** Wogonin inhibited HSV-1/2 gD mRNA transcription in HEC-1-A cells. Cells were pretreated with serial concentrations of wogonin for 30 min, and then infected with HSV-1 (HF) or HSV-2 (G) (MOI = 1). HSV-1/2 gD mRNA transcript levels were determined via qPCR 24 h p.i. **c** and **d** Wogonin suppressed HSV-1/2 gD protein expression in HEC-1-A cells **c** and Vero cells **d**. Cells were treated with wogonin prior to infection with HSV-1 or HSV-2 (MOI = 1). The gD protein expression level was determined via western blot 24 h p.i. **e** The cytotoxicity of wogonin was investigated by CCK-8 colorimetric assay after 48 h of compound exposure. All experiments were performed three times. Representative results are shown. The data are the mean values (±SDs) of triplicate determinations

Furthermore, we confirmed the anti-HSV-1/2 effect of wogonin by monitoring viral glycoprotein D (gD) expression level reductions after treatment with serial concentrations of wogonin. HSV gD is the structural component of viral envelope and a representative late (L) gene product whose levels can indicate viral protein expression and replication. As shown in Fig. 2 a and b, wogonin impeded HSV-1/2 gD messenger RNA (mRNA) transcription in a dose-dependent manner, as determined via qPCR. In parallel, gD protein expression levels were also investigated via western blot analysis to confirm the inhibitory effect of wogonin on HSV replication. Its inhibitory effect on HSV gD protein expression was consistent with the mRNA expression results obtained in both HEC-1-A and Vero cells (Fig. 2 c and d).

The cytotoxicity of wogonin was evaluated to exclude the possibility that the anti-HSV-1/2 activity of wogonin was correlated with a direct cytotoxic effect. The results shown in Fig. 2e illustrate that wogonin has low cytotoxicity toward both the HEC-1-A and Vero cell lines, with 50% cytotoxicity concentrations (CC_50_) greater than 200 μM, significantly higher than the virus inhibiting dose. These findings demonstrate that wogonin can inhibit HSV-1/2 viral replication in vitro while showing low cytotoxicity toward HSV permissive cells.

### Wogonin blocked the HSV life cycle at the postentry step

**Fig. 3:**
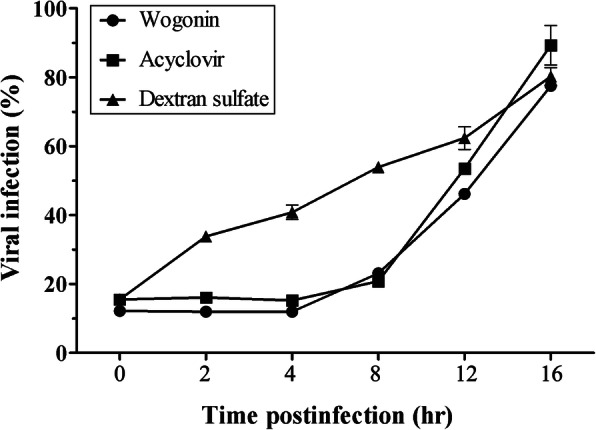
Wogonin inhibited HSV infection at a postentry step. HEC-1-A cells were infected with HSV-2 (MOI = 1) and treated with wogonin (100 μM), acyclovir (50 μg/ml) or dextran sulfate (100 μg/ml) at indicated the time points. The viral infection level is represented by gD-2 expression as determined by in-cell western assay 24 h p.i. The data represent mean values (±SDs) of triplicate determinations from three independent experiments

To explore the antiviral mechanism of wogonin, we identified the stage of the HSV viral life cycle that was blocked by wogonin. A time-of-drug-addition assay was used as a simple and clear approach to provide insight into its mode of action. As shown in Fig. 3, wogonin and two other drugs, acyclovir and dextran sulfate, were dispensed into HSV-2-infected HEC-1-A cells at different indicated time points. Acyclovir is a purine nucleoside analog of guanosine that is well-known as a highly potent inhibitor of HSV DNA polymerase [12]. Dextran sulfate is an HSV viral entry inhibitor, which can block the interaction between viral envelope proteins and host cellular receptors [13]. These two drugs with different antiviral action modes were used as the “reference drugs”. It was found that wogonin and acyclovir inhibited HSV-2 replication from 0 to 8 h p.i., while dextran sulfate, a viral attachment and entry inhibitor, did not exert inhibitory effect from 0 to 2 h p.i. The results suggest that wogonin might act after viral entry to block a certain postentry stage of the HSV life cycle.

### Wogonin impeded HSV immediate-early (IE) gene expression

**Fig. 4:**
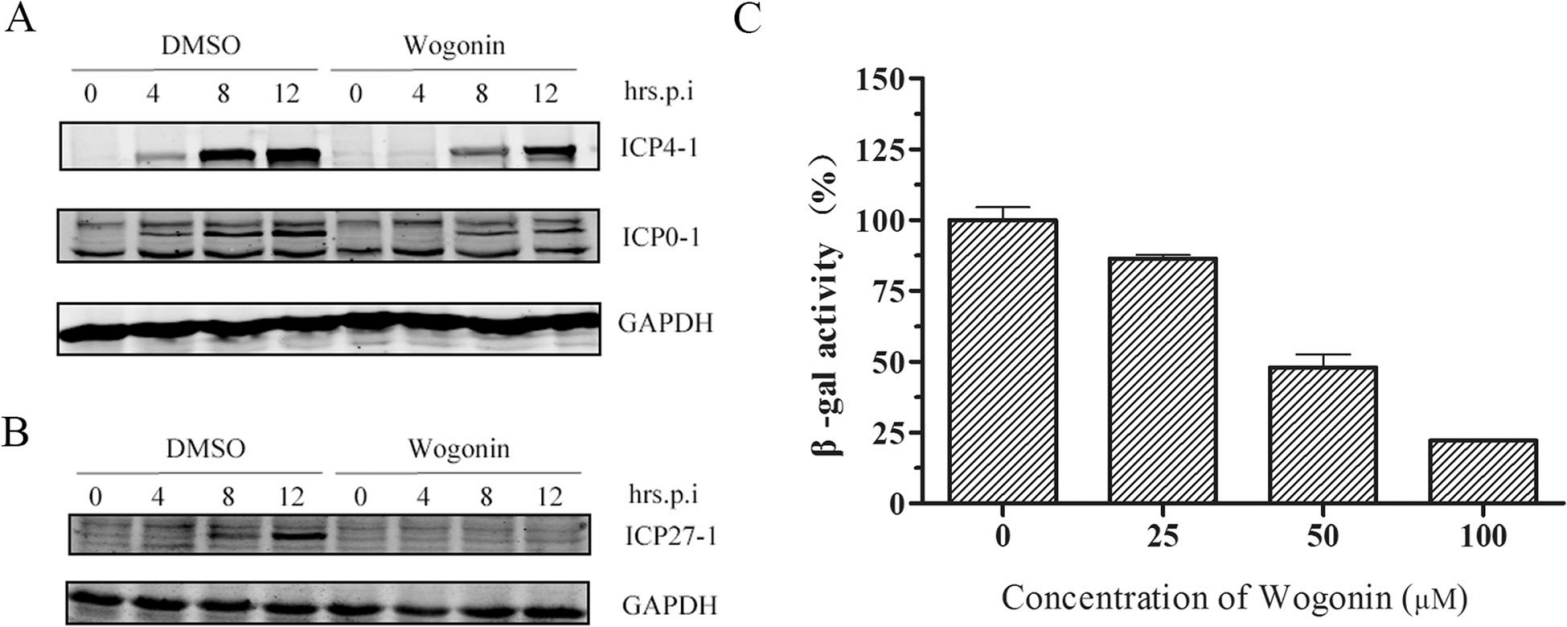
Wogonin inhibited HSV IE gene expression. **a** to **b**) HEC-1-A cells were either mock-treated or treated with wogonin (100 μM) and then infected with HSV-1 (MOI = 1). The protein expression levels of ICP4, ICP0 (A) and ICP27 **b** at each time point were determined via western blot analysis and normalized to those of GAPDH. **c** Wogonin inhibited HSV-1/blue ICP4 promoter-driven *lac*Z gene expression in a dose-dependent manner. HEC-1-A cells were pretreated with serial concentrations of wogonin 30 min prior to infection with HSV-1/blue (MOI = 1). The β-Gal activity was measured as described 12 h p.i. The data are presented as the mean values (±SDs) of triplicate determinations from three dependent experiments

HSV IE genes are key for viral gene transcription and protein expression. After entering into a host cell, IE genes, including Infected cell polypeptide 27 (ICP27), Infected cell polypeptide 4 (ICP4) and Infected cell polypeptide 0 (ICP0) act in part to upregulate viral early (E) and L genes [14]. Therefore, we also investigated the effect of wogonin on representative viral IE gene expression. ICP4 is a major viral transcription factor of HSV. Notably, wogonin suppressed ICP4 protein expression in a time-dependent manner; the results showed that ICP4 protein expression was completely inhibited at 4 h p.i. and significantly inhibited at 8 and 12 h p.i. (Fig. 4a). The effect of wogonin on ICP0 protein expression was also evaluated, and similar results were obtained in Fig. 4b. ICP27, which contributes to nuclear export of viral mRNAs, was also blocked completely by wogonin from 8 to 12 h p.i. We further employed HSV-1/blue recombinant virus with an ICP4 promoter-driven *lacZ* reporter gene to confirm our findings. As shown in Fig. 4c, wogonin inhibited ICP4 promoter-driven *lacZ* gene expression in a dose-dependent manner. It was suggested that wogonin might inhibit HSV replication by interfering with viral IE gene expression and functions.

### Wogonin attenuated HSV-2-induced NF-kB activation

**Fig. 5:**
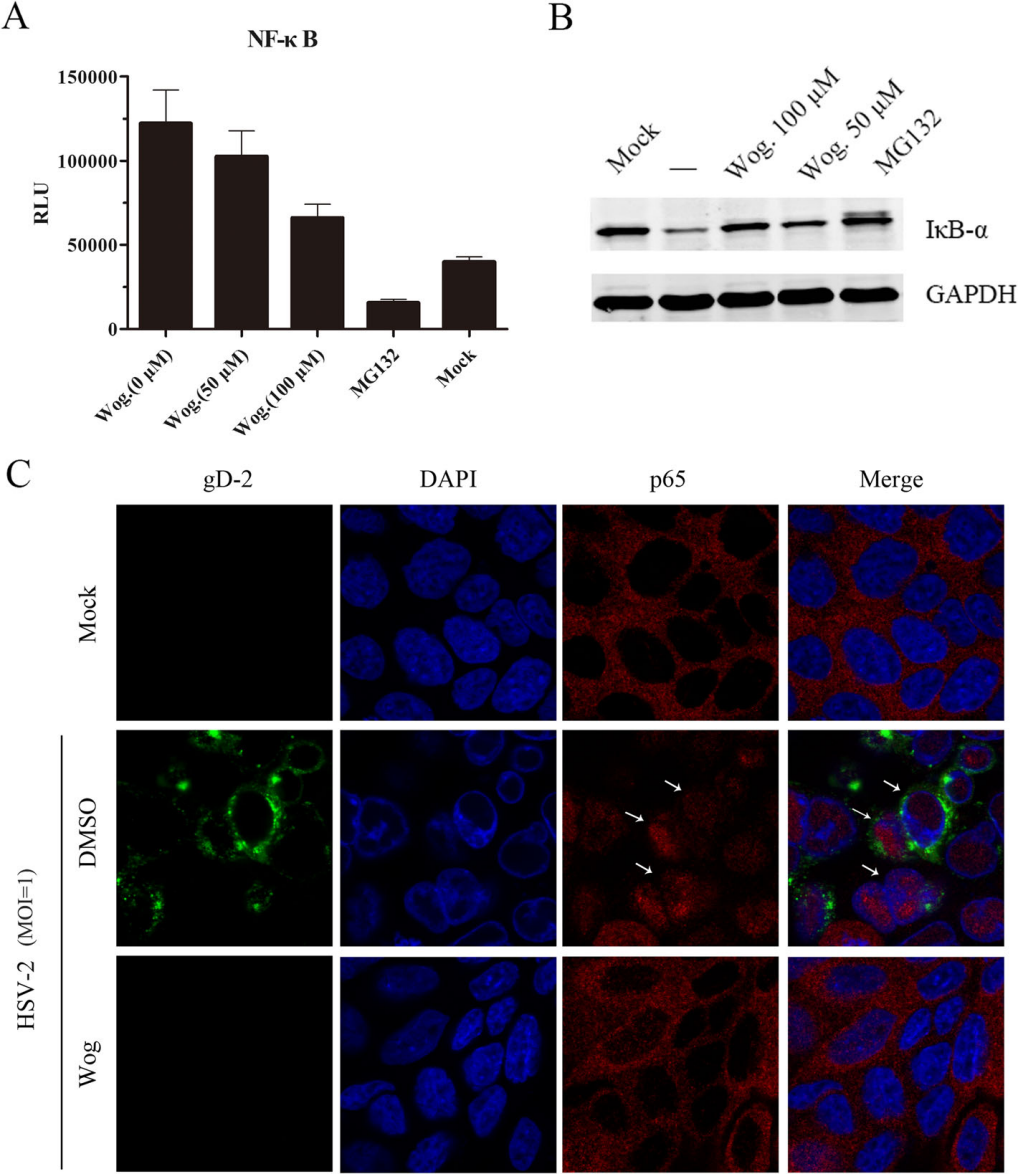
Wogonin attenuated HSV-stimulated NF-κB activation. **a** HEC-1-A cells were transfected with an NF-κB-luc reporter plasmid. The cells were mock-treated or treated with the indicated concentrations of wogonin or MG132 (5 μg/ml) prior to mock infection or infection with HSV-2 (MOI = 1). The relative luciferase activity was determined after 24 h. The data are presented the mean values (±SDs) of triplicate determinations from three dependent experiments. **b** Wogonin prevented virus-induced IκB-α degradation. HEC-1-A cells were mock-infected or infected with HSV-2 (MOI = 1) in the absence or presence of wogonin (50 and 100 μM) or MG132 (5 μg/ml). The IκB-α levels were visualized 24 h p.i. by western blot analysis. **c** Wogonin reversed HSV-2-induced p65 nuclear translocation. HEC-1-A cells were mock-infected or infected with HSV-2 (MOI = 1) in the presence or absence of wogonin (100 μM). The p65 translocation was determined via immunofluorescence assay 24 h p.i

Previous studies have demonstrated that HSV-induced persistent activation of the NF-κB pathway is a prerequisite for viral replication and host cell survival at the early stage of the HSV life cycle [15–17]. Therefore, we investigated whether wogonin influences HSV-2-induced NF-κB activation. First, we employed an NF-κB-luciferase (NF-κB-luc) reporter system. As shown in Fig. 5a, HSV-2 infection activated NF-κB response element (RE)-mediated luciferase expression significantly, and wogonin attenuated this effect in a dose-dependent manner. In contrast, the NF-κB specific inhibitor MG132 completely inhibited virus-stimulated NF-κB activation. Inhibitor kappa B-alpha (IκB-α) degradation is a distinct marker of cellular NF-κB pathway activation; thus, we also evaluated IκB-α levels in HSV-2-infected HEC-1-A cells that were left untreated or were treated with wogonin or MG132. As shown in Fig. 5b, HSV-2-stimulated IκB-α degradation was blocked by wogonin and MG132. In addition, p65 nuclear translocation, which is often used as an indicator of NF-κB activation, was investigated after viral infection via confocal imaging. As shown in Fig. 5c, Wogonin simultaneously inhibited HSV-stimulated p65 nuclear translocation and viral gD expression in HEC-1-A cells. Taken together, we concluded that wogonin could inhibit HSV-2-induced NF-κB activation and result in inhibition of HSV IE gene expression and replication.

### Wogonin suppressed HSV-2-induced MAPK activation

**Fig. 6:**
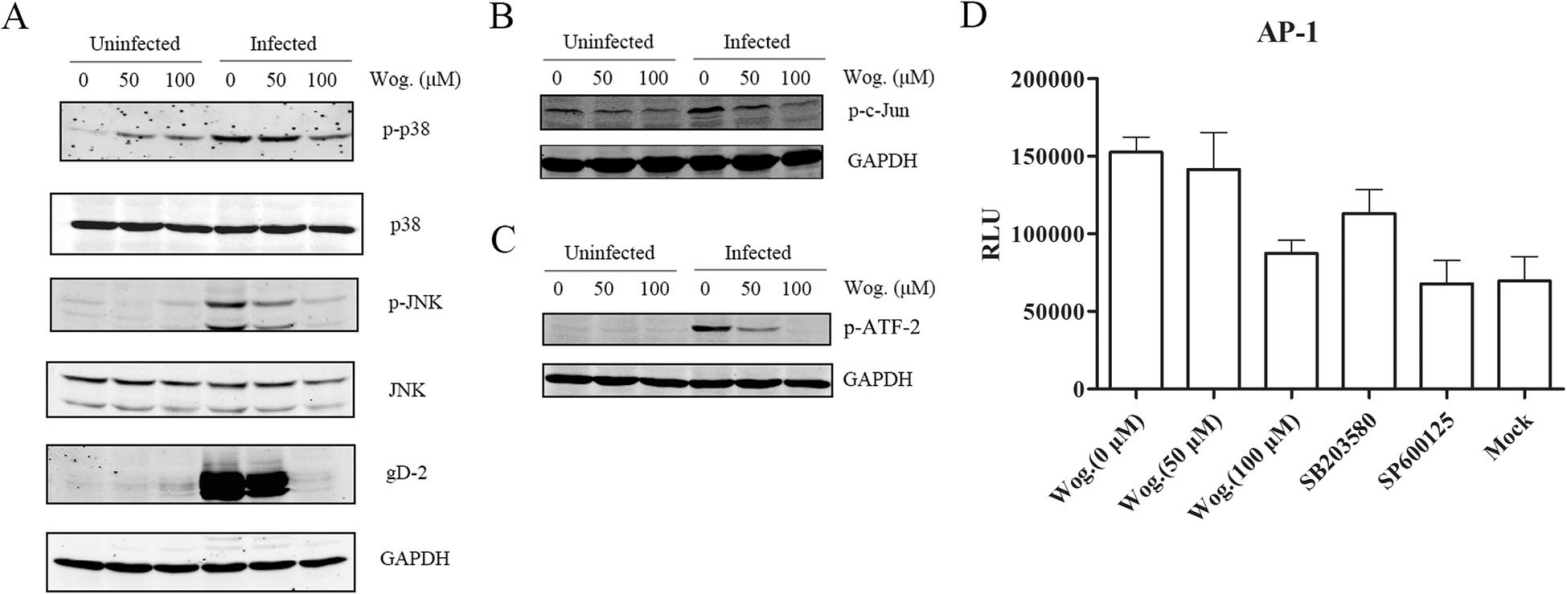
Wogonin inhibited HSV-2-induced JNK and p38 MAPK activation. **a** to **c** Wogonin inhibited HSV-2-induced JNK and p38 MAPK phosphorylation. HEC-1-A cells were mock-infected or infected with HSV-2 (MOI = 1) in the presence or absence of wogonin (100 μM). The levels of JNK, p38 MAPK, their phosphorylated forms and the downstream factors p-c-Jun andp-ATF-2 were determined by western blot 24 h p.i. **d** Wogonin inhibited HSV-2-induced AP-1 activation. HEC-1-A cells were transfected with an AP-1-luc reporter plasmid and then the cells were mock-treated or treated with serial concentrations of wogonin, SB203580 (20 μM) or SP600125 (20 μM) prior to mock infection or infection with HSV-2 (MOI = 1). SB203580 and SP600125, which are p38/MAPK and JNK inhibitors, respectively, were used as the positive controls. The relative luciferase activity was determined after 24 h. The data are presented as mean values (±SDs) of triplicate determinations from three independent experiments

Cellular JNK and p38 MAPK pathways are required for HSV viral protein expression and facilitate the viral replication [18, 19]. Our earlier work has also verified that JNK and p38 MAPK pathways can be stimulated by HSV-2 infection in HEC-1-A cells [20]. Therefore, we investigated the inhibitory effect of wogonin on the activation of these two pathways. The results showed that wogonin attenuated the phosphorylation of p38 and JNK stimulated by viral infection (Fig. 6a). The phosphorylation of c-Jun and ATF-2 was also studied. c-Jun is a downstream substrate of JNK, and ATF-2 is a common substrate of both JNK and p38 MAPK. The phosphorylation of c-Jun and ATF-2 was observed after HSV-2 infection, and wogonin suppressed these two markers (Fig. 6b and c). An AP-1-luciferase (AP-1-luc) reporter system was utilized to evaluate whether wogonin can inhibit HSV-2-induced activation of AP-1, which is a major downstream transcription factor of the JNK/p38 MAPK pathway. As shown in Fig. 6d, wogonin suppressed AP-1 activation in a dose-dependent manner, and two inhibitors, SP600125 (a potent JNK antagonist) and SB203580 (a potent p38 antagonist), also attenuated HSV-2-stimulated AP-1 activation. Thus, wogonin might attenuate HSV-2-induced JNK/p38 MAPK activation to interfere with viral replication.

### Synergistic effects of wogonin and acyclovir against HSV-2

**Fig. 7:**
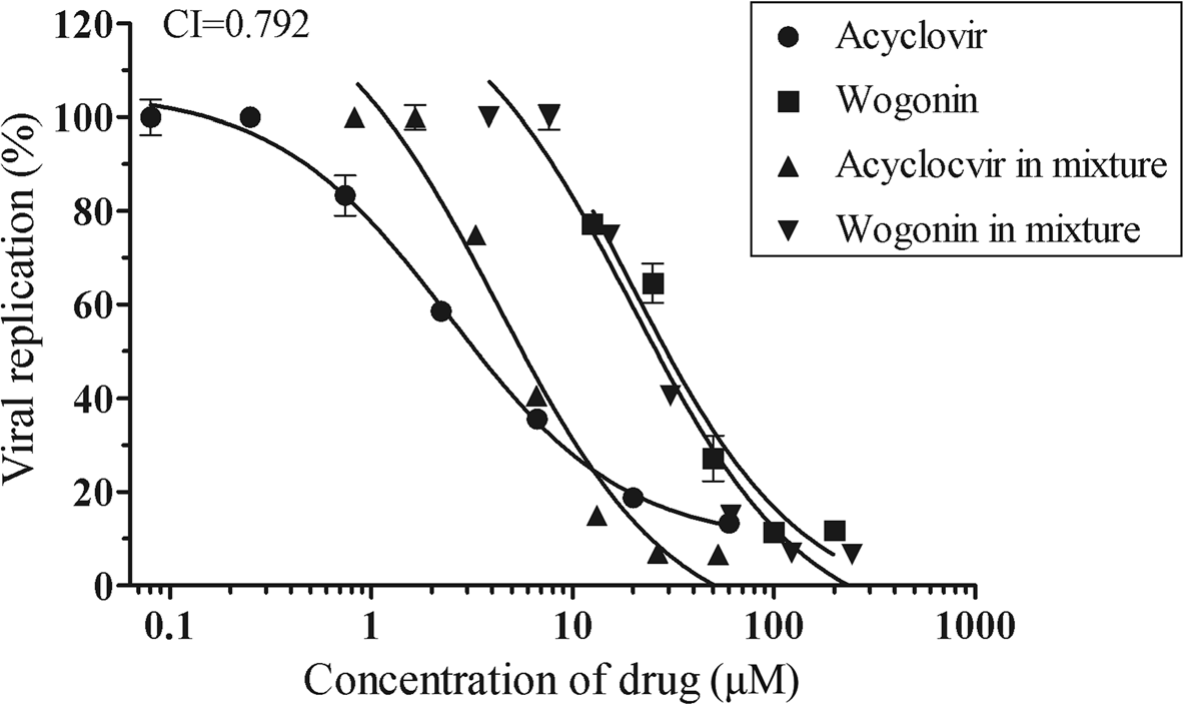
Wogonin exerted a moderate synergistic effect with acyclovir against HSV-2 infection. The effective HSV-2 infection-inhibiting concentrations of the compounds alone and in combination are plotted in two curves. The CI values were calculated using CalcuSyn software and evaluated as described. The data are presented as the mean values (±SDs) of triplicate determinations from three independent experiments

Combined treatments with drugs that exert synergistic effects have potential for clinical applications. To evaluate the synergism of wogonin and acyclovir, the anti-HSV-2 activity of the two drugs individually and in combination was determined by in-cell western assay. As shown in Fig. 7, the combination index (CI) was 0.792, which indicates that wogonin and acyclovir exhibit moderate synergism when used in combination. This finding suggests a potential beneficial effect of combined wogonin and acyclovir treatment.

## Discussion

Traditional Chinese herbal medicines have long been used to prevent and treat viral infectious diseases in China and other oriental countries and are an important sources for antiviral agent discovering [21]. Wogonin, a main pharmacological ingredient in *Scutellaria* radix, exerts inhibitory effects on some human viruses, including RSV, HBV and VZV [8–11]. In this study, we found that wogonin can block HSV-1 or HSV-2 viral replication and protein expression in vitro. Further results demonstrated that wogonin acted as a postentry inhibitor and probably affected the step between viral entry and viral genomic DNA replication. In the HSV life cycle, IE, E and L genes are expressed after transport of viral DNA into the nucleus. The protein expression of IE gene is a prerequisite for viral E and L gene transcription and expression and play a vital role in the whole viral life cycle. The results illustrated that wogonin attenuated the protein expression of ICP0, ICP4 and ICP27. Our findings suggested that wogonin suppresses HSV IE gene expression, interfering with HSV downstream E and L gene expression.

We hypothesized that certain host cellular pathways affect viral IE gene promoter transcriptional activity and thus influence viral E and L gene expression. We thus studied the effects of wogonin on host cellular signaling pathways and found that wogonin suppressed HSV-2-stimulated NF-κB pathway activation, suppressing IκB-α degradation and p65 nuclear translocation. Previous studies have shown that NF-κB is necessary for prevention of host cell apoptosis during the early stage of HSV infection [15, 22]. Additionally, NF-κB is a key regulator of cellular events including immune modulation and inflammatory and antiapoptotic responses [23]. In fact, the NF-κB pathway is also important for the invasion and infection of certain pathogens. Many human viruses have evolved to utilize the host cellular NF-κB pathway, such as HBV [24], hepatitis C virus [25], HIV-1 [26], RSV [27] and Epstein-Barr virus [28]. HSV has also been reported to activate the NF-κB pathway to facilitate its replication [29, 30]. Given the role of the host cellular NF-κB signaling pathway in HSV viral replication, modulation of this pathway seems to be an alternative approach to prevent viral infection [31].

Aside from the interaction between HSV and the NF-κB signaling pathway, interactions between certain viruses and MAPK pathway may also participate in the viral life cycle during infection. For example, a previous study has shown that rotavirus can stimulate JNK/p38 signaling pathways in permissive cell lines and enhance viral replication [32]. VZV has also been reported to stimulate JNK/p38 MAPK signaling pathways, and this pathway activation is correlated with viral replication and gene expression [33]. Mclean et al. and Zachos et al. have reported that HSV-induced JNK/p38 MAPK pathway activation leads to activation of the expression of a series of cellular genes, further helping to activate viral transcription and DNA replication [18, 19]. In this study, wogonin inhibited AP-1 activation in a dose-dependent manner and downregulated HSV-induced phosphorylation of c-Jun and ATF-2, the two components of the transcription factor AP-1. Although wogonin slightly increased the levels of phosphorylated p38 in mock-infected cells, it inhibited HSV-induced p38 phosphorylation in virus infected cells (Fig. 6a). In addition, wogonin suppressed HSV-induced JNK phosphorylation in a dose-dependent manner. Based on these observations, we postulate that wogonin may act as an inhibitor of HSV-induced JNK/p38 MAPK activation and thus of HSV infection.

Previous studies have shown that combination treatment with acyclovir and another drug with a different antiviral mechanism may increase anti-HSV activity in vitro and in vivo [34, 35]. Our data demonstrate that wogonin has a moderate synergistic effect with acyclovir against HSV-2 replication in vitro (Fig. 7). Therefore, combination treatment with wogonin and acyclovir may have therapeutic potential for HSV infection.

## Conclusions

In this study, we investigated the antiviral mechanism of wogonin in the context of HSV-1/2 infection and found that wogonin significantly suppressed HSV-2-induced NF-κB and JNK/p38 MAPK activation. Thus, we conclude that the inhibitory effect of wogonin on these signaling pathways may account for the anti-HSV activity of this compound. However, further study is required to clarify the correlation between IE genes and HSV-induced signaling pathways in detail. Given its low cytotoxicity and long history of medical use, wogonin may be a potential antiherpes drug candidate worthy of further study as an alternative treatment.

## Methods

### Reagents, cell lines, plasmids and viruses

Wogonin and acyclovir were obtained from the National Institutes for Food and Drug Control in China (Beijing, China). SB203580, SP600125, and MG132 were purchased from Beyotime Biotechnology Institute (Haimen, Jiangsu, China). Alexa Fluor 488-conjugated goat anti-mouse IgG (H + L), DAPI, DRAQ5 and SYBR green real-time PCR reagent were obtained from Life Technologies, Thermo Fisher Scientific (Carlsbad, CA, USA). IRDye 680-conjugated goat-anti-rabbit and IRDye 800-conjugated goat-anti-mouse antibodies were obtained from LI-COR (Lincoln, NE, USA). Antibodies specific for HSV-1/2 gD, HSV-1 ICP0, HSV-1 ICP4, HSV-1 ICP27, JNK2, p38, GAPDH, and RIPA lysis buffer were purchased from Santa Cruz (Santa Cruz, CA, USA). p65, p-p38, p-c-Jun, p-JNK1/2, p-ATF-2, and IκB-α antibodies were purchased from Cell Signaling Technology (Beverly, MA, USA). Bright-Glo luciferase assay system was purchased from Promega (Madison, WI, USA).

Vero and HEC-1-A cells were obtained from the American Type Culture Collection (ATCC, Manassas, VA, USA). NF-κB-luc and AP-1-luc reporter plasmids were purchased from Clontech (Palo Alto, CA, USA). HSV-1(HF), HSV-1/blue and HSV-2 (G) were propagated and titrated in Vero cells as described previously [36].

### In vitro viral inhibition assay

The in vitro viral inhibitory effects of wogonin were determined via titration of infectious virions according to a previously described method [37]. Briefly, confluent HEC-1-A cells in 96-well plates were pretreated with serial concentrations of wogonin for 30 min and then infected with HSV-1 or HSV-2 (MOI = 1). At 24 h p.i., the culture medium on treated HEC-1-A cells was replaced with fresh medium. The infected cells were frozen and thawed in 3 cycles to release the virions. The virions-containing medium was dispensed on confluent Vero monolayer cells, and the viral titration was performed by counting the numbers of plaques after 48 h.

### In vitro cytotoxicity assay

In vitro cytotoxicity was determined using a commercial Cell Counting Kit-8 (CCK-8, Dojindo, Kumamoto, Japan) according to the manufacturer’s instructions. Briefly, cells were seeded at a density of 2 × 10^4^ cells per well into 96-well plates, cultured for 24 h, and then treated with serial dilutions of compound in triplicate. After 24 h of incubation, 10 μl of CCK-8 reagent was added to each well, and the plates were incubated at 37 °C for 3 h. The absorbance at 450 nm was measured using a Tecan Infinite M200 microplate reader (Männedorf, Switzerland), and cell viability was plotted as the percentage of viable cells compared with that of the mock-treated control cells.

### Western blot and in-cell western assays

Western blot and in-cell western assays were performed as described previously [20]. Cells were lysed using RIPA lysis buffer on ice for 30 min and then centrifuged at 12,000×g for 10 min at 4 °C to collect the supernatants. The total protein concentrations were determined using a BCA protein assay kit (Thermo Fisher Scientific, Waltham, MA, USA). After separation via SDS-PAGE, the proteins were electrotransferred onto polyvinylidene difluoride (PVDF) membranes (Millipore, Billerica, MA, USA), The membrane were blocked using Odyssey blocking buffer (LI-COR) and primary and incubated with secondary antibodies, and the protein bands were visualized via an Odyssey Infrared Imager (LI-COR).

An in-cell western assay was performed in a 96-well plate. Cells were fixed with 4% paraformaldehyde for 20 min at room temperature (RT) and permeabilized via five washes in 0.1% Triton X-100 in phosphate-buffered saline (PBS) for 5 min per wash. The cell monolayers were blocked for 90 min in blocking buffer consisting of4% nonfat dry milk in PBS with 0.1% Tween-20 (PBS-T) and then incubated with primary antibodies diluted in blocking buffer (1:200) for 2 h at RT. After washing with PBS-T, the cell layers were stained with IRDye IgG (1:1500) for 1 h. The plate was rinsed and scanned in an Odyssey Infrared Imager. The relative protein expression levels were normalized to those of DRAQ5.

### Time-of-drug-addition assay

A time-of-drug-addition assay was carried out via measurement of HSV-2 gD protein expression levels, which can indicate viral replication efficiency. HEC-1-A cells were seeded into a 96-well plate and infected with HSV-2 (MOI = 1). Wogonin or other drugs with known inhibitory mechanisms were dispensed at different time points. The viral gD levels were determined via in-cell western assay at 24 h p.i. as described previously [38].

### Cell transfection and luciferase assays

HEC-1-A cells were transiently transfected with luciferase reporter plasmids using Lipofectamine 2000 transfection reagent (Life Technologies, Thermo Fisher Scientific). The relative luminescence units (RLUs) were determined using a Bright-Glo luciferase assay system (Promega). Briefly, HEC-1-A cells were seeded into 96-well plates. When the confluence reached ~ 90%, the cells were transfected with 100 ng of NF-κB or AP-1 luciferase reporter plasmid. The cells were subsequently cultured for 24 h and then treated with inhibitors for another 24 h. The luminescence signals were monitored with GloMax-96 microplate luminometer (Promega).

### RNA extraction and quantitative PCR

Total RNA was extracted using TRIzol reagent (Life Technologies, Thermo Fisher Scientific) according to the manufacturer’s instructions. Complementary DNA (cDNA) was reverse-transcribed using a ReverTra Ace qPCR RT kit (Toyobo, Osaka, Japan). Real-time qPCR was performed in triplicate on an ABI Prism 7300 Sequence Detection System using SYBR Green PCR Master Mix (Life Technologies). The sequences of the primers used in this study are as follows: HSV-1 gD, 5′-AGCAGGGGTTAGGGAGTTG-3′ (Forward) and 5′-CCATCTTGAGAGAGGCATC-3′ (reverse); HSV-2 gD, 5′- CCAAATACGCCTTAGCAGACC-3′ (forward) and 5′-CACAGTGATCGGGATGCTGG-3′ (reverse); human GAPDH, 5′-TGCACCACCAACTGCTTAGC-3′ (Forward) and 5′- GGCATGGACTGTGGTCATGAG-3′ (reverse). The mRNA transcription levels were standardized against those of the housekeeping gene GAPDH.

### HSV-1/blue assay

An HSV-1/blue assay was performed as previously described with modifications [39]. The confluent HEC-1-A cells in a 96-well plate were preincubated with serial dilution of drugs for 30 min at 37 °C. The cells were then infected with HSV-1/blue (MOI = 1). The cells were lysed with 1% NP-40 in DMEM 12 h p.i. Cell lysates from each well were then transferred into a new Costar 96-well flat plate. The β-gal substrate solution chlorophenol red-β-D-galactopyranoside (CPRG) was added to each well. The absorbance at 570 nm was measured after 1 h using a Tecan Infinite M200 microplate reader.

### Immunofluorescence staining and confocal microscopy

HEC-1-A cells were seeded onto 10 mm glass coverslips, which were then placed in a 24-well plate. The cells growing on the coverslips were rinsed with PBS, fixed with 4% paraformaldehyde for 15 min at RT, and permeabilized with 0.2% Triton X-100 for 15 min. The coverslips were blocked with 1% BSA in PBS for 30 min at RT. Target biomarkers were immunolabeled using appropriate primary antibodies and Alexa Fluor 488-conjugated secondary antibody. Nuclei were visualized by staining with DAPI. Images were acquired using an Olympus FluoView FV10i confocal microscope (Tokyo, Japan).

### Drug synergism analysis

We employed an in-cell western assay to determine the efficiency of wogonin- and acyclovir-mediated inhibition of HSV-2 gD expression in HEC-1-A cells. The half maximal effective concentration (EC_50_) of the two compounds were calculated. Combinations of the compounds with fixed molar ratios were also investigated. The ratio was optimized to yield the greatest synergism over a range of serial dilutions. The combination index (CI) of the two drugs was calculated in CalcuSyn software (Biosoft, Cambridge, UK) with a method described by Chou and Talalay [40]. Synergy was assessed according to the CI values and scored as follows: CI < 0.1, very strong synergism; CI = 0.1 to 0.3, strong synergism; CI = 0.3 to 0.7, synergism; CI = 0.7 to 0.85, moderate synergism; CI = 0.85 to 0.90, slight synergism; CI = 0.9 to 1.1, nearly additive synergism; and CI = > 1.1, antagonism.

## Supplementary information

**Additional file 1.**

## Data Availability

The datasets used and/or analyzed during the current study are available from the corresponding author on reasonable request.

## Abbreviations

AP-1: Activator protein 1
ATF-2: Activating transcription factor 2
DAPI: 4′,6-diamidino-2-phenylindole
DHBV: Duck hepatitis B virus
GAPDH: Glyceraldehyde-3-phosphate dehydrogenase
HBV: Hepatitis B virus
