# Supplementary material for: Wogonin inhibits in vitro herpes simplex virus type 1 and 2 infection by modulating cellular NF-κB and MAPK pathways
Source: BMC Microbiol. 2020 Jul 28;20:227. doi: 10.1186/s12866-020-01916-2 (PMC7388529; doi:10.1186/s12866-020-01916-2)

uncropped blots related to Fig.2

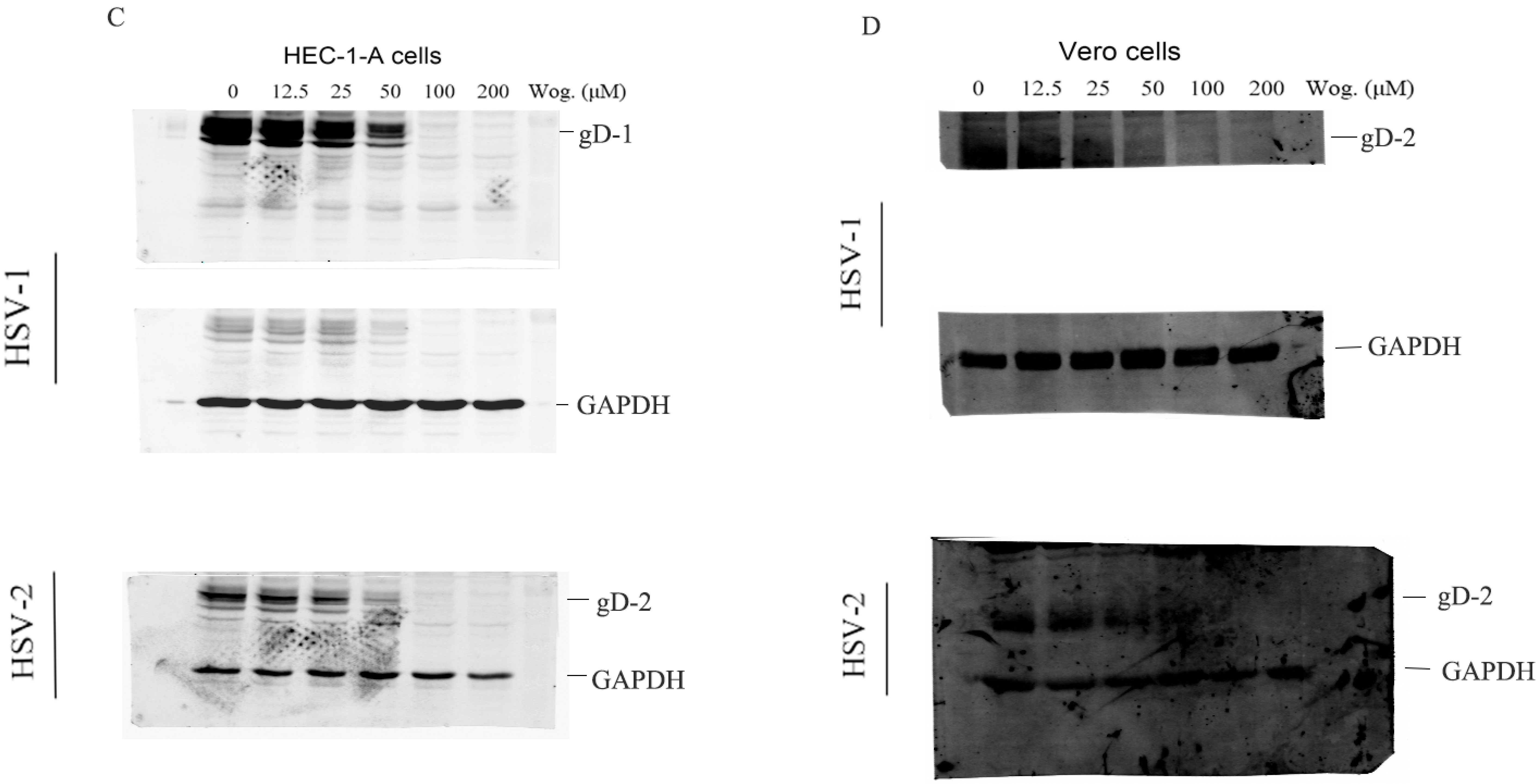

uncropped blots related to Fig.4

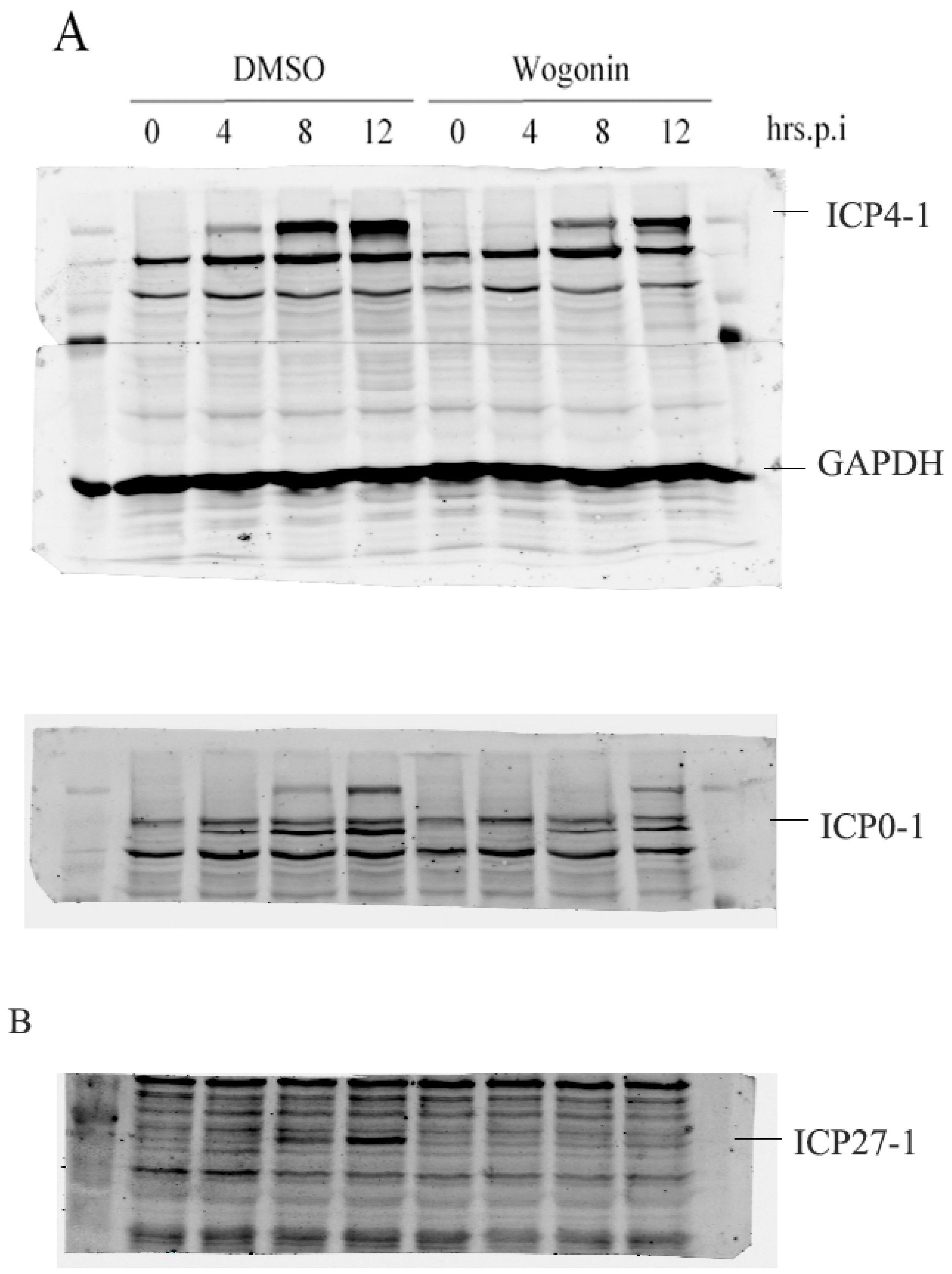

uncropped blots related to Fig.5

B

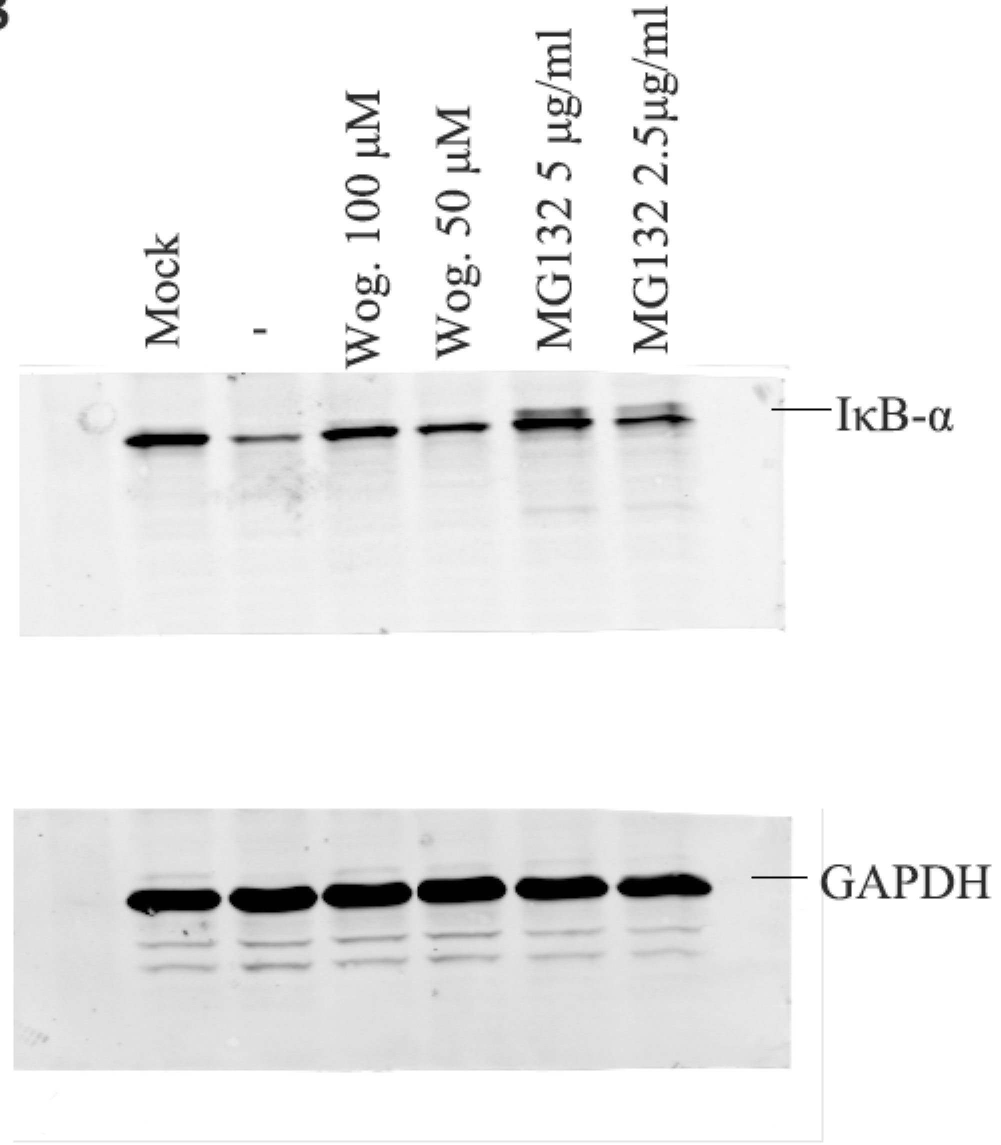

uncropped blots related to Fig.6

A

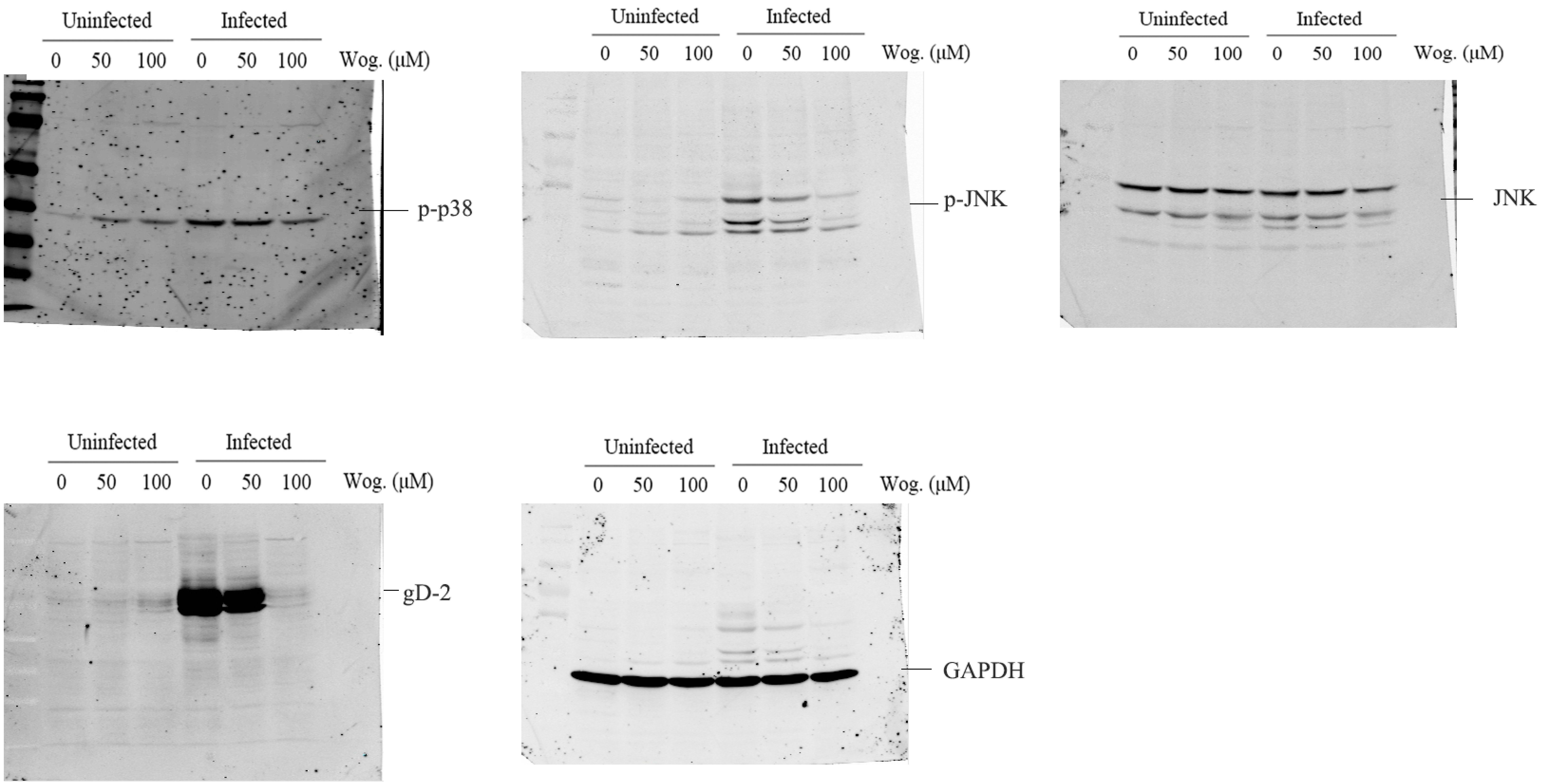

B

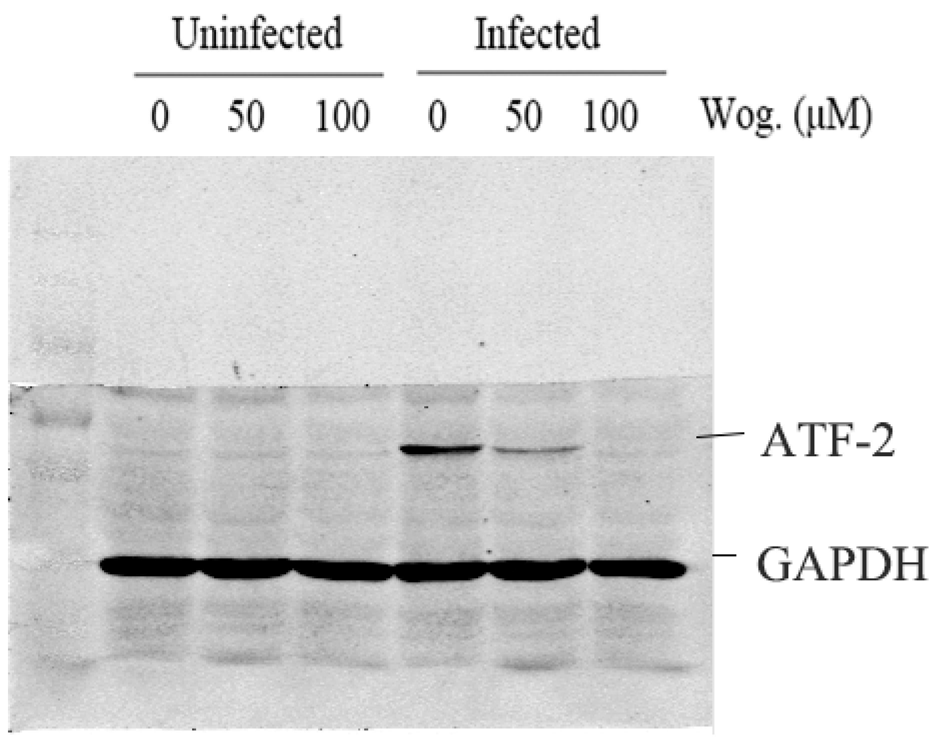

C

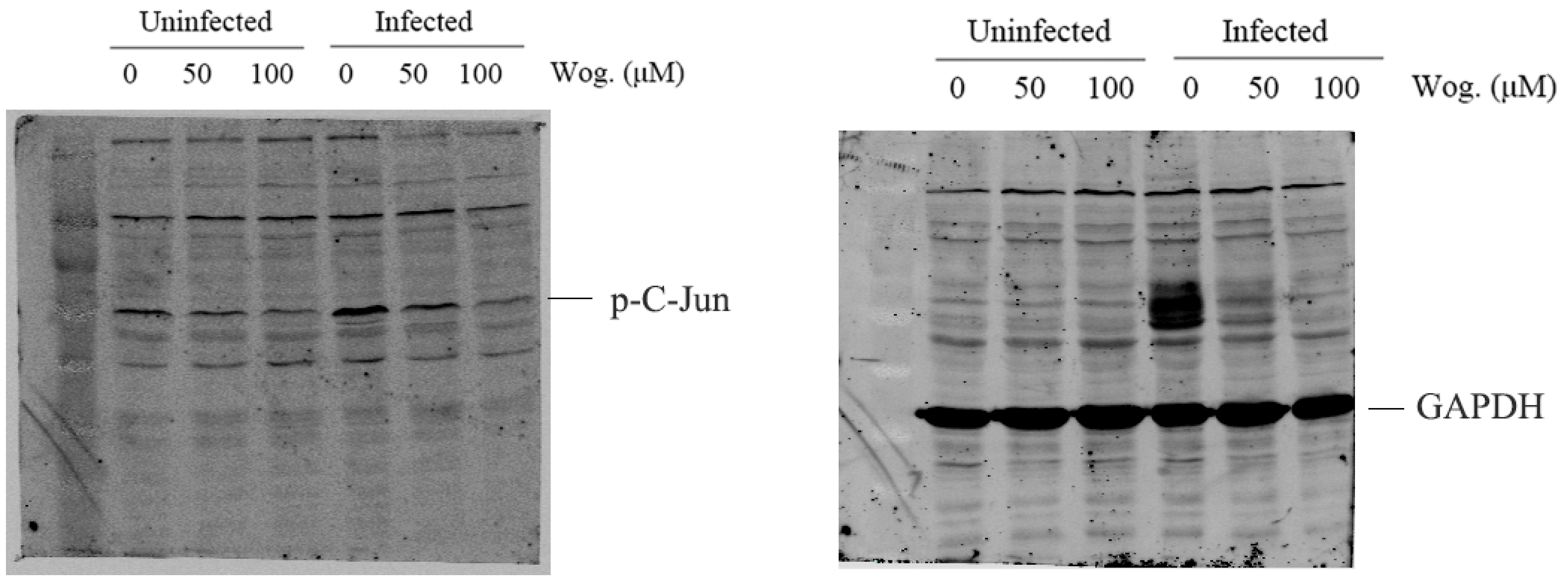

Supplement: Supplementary file 1 — Additional file 1. [file 12866_2020_1916_MOESM1_ESM.pdf]
